# Honey in wound healing: molecular and cellular mechanisms of inflammation control, immune modulation, and antimicrobial defense

**DOI:** 10.1515/biol-2025-1397

**Published:** 2026-10-30

**Authors:** Abdalsalam Kmail, Bashar Saad

**Affiliations:** Faculty of Sciences, Arab American University, P.O. Box 240, Jenin, Palestine; Faculty of Medicine, Arab American University, P.O. Box 240, Jenin, Palestine; Qasemi Research Center and Faculty of Medicine, Arab American University, P.O. Box 240, Jenin, Palestine

**Keywords:** honey, wound healing, inflammation, oxidative stress, antimicrobial activity

## Abstract

Wound healing is a complex process involving inflammation control, protection against infection, oxidative balance, and tissue regeneration. Honey has attracted considerable attention due to its multifunctional biological activities. In addition to its anti-inflammatory and antioxidant actions, honey exhibits broad antimicrobial activity that helps prevent microbial colonization and biofilm formation, thereby facilitating a favorable environment for healing. It also limits oxidative stress through reactive oxygen species (ROS) scavenging and erythroid 2–related factor 2 (Nrf2) activation, resulting in better control of inflammation and improved tissue repair. However, despite promising experimental and clinical evidence, the clinical translation of honey remains limited by variability in composition, lack of standardization, and methodological weaknesses in existing trials, and robust, well-controlled studies and standardized formulations are therefore essential to confirm efficacy and support its integration into evidence-based wound care. This review summarizes current knowledge on how honey supports wound healing, focusing on its antimicrobial, anti-inflammatory, antioxidant, and immunomodulatory effects. The analysis is based on a literature search of MEDLINE, Google Scholar, Scopus, and Web of Science. Overall, honey reduces pro-inflammatory mediators (e.g., tumor necrosis factor-α (TNF-α), interleukin (IL)-1β, IL-6, nuclear factor kappa B (NF-κB)) and enhances anti-inflammatory responses such as IL-10 and M2 macrophage activity.

## 1 Introduction

A wound is a disruption of the normal anatomical structure and functional integrity of the skin and, depending on its depth and severity, may extend into the underlying tissues. Wounds can be acute, resulting from trauma, burns, or surgical procedures, or chronic, as occurs in conditions such as diabetic foot ulcers (DFUs), venous leg ulcers (VLUs), and pressure injuries (PIs). Effective wound healing requires the coordinated progression of hemostasis, inflammation, proliferation, and tissue remodeling. Disruption of this process may result in delayed healing, persistent tissue damage, infection, and progression to a chronic wound [1], 2].

Chronic wounds represent a major clinical and socioeconomic burden and remain difficult to manage because several pathological processes frequently occur simultaneously. These include persistent inflammation, excessive oxidative stress, impaired angiogenesis, defective extracellular matrix remodeling, reduced cellular proliferation and migration, microbial colonization, and biofilm formation [3], [4], [5]. Biofilms are particularly challenging because they can increase microbial tolerance to antimicrobial agents and host immune responses, thereby contributing to persistent infection and delayed tissue repair [4], 5]. In addition, underlying conditions such as diabetes, vascular insufficiency, and prolonged pressure may further compromise tissue perfusion and regenerative capacity. Consequently, effective wound management often requires simultaneous control of microbial burden, inflammation, oxidative damage, and impaired tissue regeneration. The increasing prevalence of antimicrobial resistance has further stimulated interest in naturally derived interventions with multiple biological activities [6].

Honey has been used as a wound remedy for thousands of years and represents one of the oldest naturally derived substances with documented therapeutic applications in wound care [7]. Its use has been described in ancient Egyptian medicine, including the Ebers Papyrus, as well as in Greek, Roman, Islamic, and other traditional medical systems [8], 9]. In the Greco-Arab and Islamic medical tradition, Ibn Sina (Avicenna, 980–1037) described therapeutic applications of honey and honey-containing preparations for various medical conditions, including wound-related disorders [9]. These historical observations were based largely on empirical experience; however, modern research has increasingly provided biochemical, microbiological, and cellular explanations for some of the therapeutic properties attributed to honey.

The contemporary interest in honey for wound management is based on its multifunctional biological activity. Honey possesses antimicrobial properties arising from several complementary mechanisms, including its high sugar concentration and associated osmotic effects, acidic pH, hydrogen peroxide generation, and the presence of phenolic and other phytochemical constituents [10], 11]. These properties can inhibit a broad range of wound-associated microorganisms, including *Staphylococcus aureus*, methicillin-resistant *S. aureus* (MRSA), and *Pseudomonas aeruginosa* [11]. Honey has also demonstrated activity against microbial biofilms, which is particularly relevant to chronic wounds in which biofilm-associated microorganisms may contribute to persistent inflammation and delayed healing [4], 12].

The potential role of honey in wound healing extends beyond antimicrobial activity. Honey contains sugars as its major constituents together with smaller amounts of proteins, enzymes, amino acids, minerals, vitamins, organic acids, phenolic acids, flavonoids, and other bioactive compounds [13]. These constituents may contribute to antioxidant, anti-inflammatory, immunomodulatory, and tissue-regenerative effects [10], [14], [15], [16]. Experimental studies suggest that honey can modulate inflammatory responses, reduce excessive oxidative stress, and influence cellular processes involved in tissue repair. In wound-relevant experimental models, honey and its constituents have been associated with keratinocyte migration, fibroblast proliferation and migration, extracellular matrix formation, and other processes involved in tissue regeneration [16]. Thus, honey may act on several interconnected components of the wound environment rather than functioning solely as an antimicrobial agent.

Importantly, however, honey should not be considered a chemically or biologically uniform therapeutic substance. Its composition and biological activity vary according to botanical origin, geographical location, environmental conditions, bee species, harvesting, storage, and processing [13], 17]. More than 200 compounds have been identified in honey, although their concentrations and relative contribution to biological activity vary considerably among honey types. Sugars constitute the predominant fraction, whereas minor constituents such as phenolic acids, flavonoids, enzymes, and other phytochemicals may contribute substantially to antioxidant, antimicrobial, and anti-inflammatory activity [13], 17]. Consequently, biological findings obtained with one honey cannot automatically be extrapolated to all other honeys.

This compositional diversity is particularly important when considering honey for wound management. Several monofloral and geographically distinctive honeys have been investigated, including Manuka, Tualang, Gelam, Sidr, Tamarix, Eucalyptus, and *Medicago sativa* honeys, among others. Manuka honey, derived predominantly from Leptospermum species, is among the most extensively studied and is characterized by non-peroxide antibacterial activity associated particularly with methylglyoxal and other constituents [18]. Tualang, Gelam, Sidr, Tamarix, Eucalyptus, and *M. sativa* honeys contain different combinations and concentrations of phenolic acids, flavonoids, enzymes, and other compounds that may contribute to differences in their biological activities [19]. Recent research further demonstrates that even commercially available medical-grade honeys of different botanical origins can display distinct phytochemical profiles, antioxidant capacities, hydrogen peroxide production, antibacterial activity, and effects on fibroblast migration, supporting the concept that honey type is an important determinant of biological activity [20], [21], [22], [23], [24].

Specific phytochemicals may also contribute to the biological differences among honeys. For example, chlorogenic acid and other phenolic compounds identified in Tamarix honey have been associated with antioxidant and wound-relevant biological effects [14], whereas phenolic constituents such as ellagic acid have been reported in *M. sativa* honey and may contribute to antioxidant and anti-inflammatory activities [15]. Such observations provide a rationale for linking the botanical origin and chemical composition of individual honeys with their biological effects rather than treating honey as a single standardized natural product.

Honey has been investigated for several wound categories, including burns, infected wounds, surgical and traumatic wounds, diabetic ulcers, venous ulcers, and other chronic wounds [12], [13], [14], [15], [16, 25]. However, the strength of evidence varies substantially according to honey type, formulation, wound category, study design, and outcome measure. Clinical evidence is most developed for medical-grade honey preparations and selected wound types, whereas many monofloral honeys have been evaluated predominantly *in vitro* or in animal models. Recent systematic evidence suggests that honey dressings may improve healing outcomes in chronic wounds, but the certainty of evidence remains limited by heterogeneity, risk of bias, and differences among interventions [26]. Similarly, recent evidence in diabetic foot ulcers suggests potential improvements in healing rate and time to healing, although the quality of evidence is not uniformly high [27]. These findings emphasize the need to distinguish between promising biological activity and established clinical efficacy.

Another important consideration is the distinction between food-grade honey and medical-grade honey (MGH). Raw or food-grade honey should not automatically be regarded as equivalent to a standardized therapeutic product. Medical-grade honey is produced and processed under controlled conditions intended to minimize contamination and ensure reproducible physicochemical and microbiological characteristics. Current proposals for MGH standards emphasize controlled production, sterilization, safety criteria, physicochemical characterization, and demonstrated biological activity [28], 29]. This distinction is essential when translating experimental findings into clinical wound care.

Advances in biomaterial engineering have further expanded the potential applications of honey. Honey has been incorporated into hydrogels, films, hydrocolloid dressings, nanoparticles, and biopolymeric scaffolds to improve wound-site retention, moisture control, controlled release, and interaction with the wound environment [2], 25]. Such formulations may help overcome some limitations associated with the direct topical application of honey and provide opportunities for combining its biological activity with modern wound-dressing technologies.

Despite the increasing evidence supporting honey-based wound therapies, several important questions remain unresolved. Considerable variability exists in the botanical origin and chemical composition of honeys, while studies frequently use different honey concentrations, preparation methods, wound models, microbial strains, formulations, and outcome measures [17], 26]. These differences make direct comparison difficult and limit the generalization of findings from one honey type to another. Moreover, although numerous *in vitro* and animal studies demonstrate antimicrobial, antioxidant, anti-inflammatory, or regenerative effects, clinical evidence remains more limited and heterogeneous for many specific honey varieties. The relationship between the chemical composition of individual honey types and their specific wound-healing effects therefore requires further clarification.

Accordingly, this review does not consider honey as a single, uniform natural remedy. Instead, it examines the relationship between honey type, botanical origin, chemical composition, biological mechanism, wound type, and level of evidence. Particular attention is given to selected honeys for which wound-related biological or clinical evidence is available, including their antimicrobial and antibiofilm, antioxidant, anti-inflammatory, immunomodulatory, and pro-regenerative properties. The emphasis on immunomodulation is particularly relevant because successful wound healing depends on the timely activation, coordination, and resolution of innate and adaptive immune responses, whereas persistent or dysregulated inflammation is a major characteristic of chronic wounds. Honey may influence this inflammatory environment not only by reducing microbial burden and oxidative stress but also by modulating immune-cell activity and inflammatory mediators, thereby potentially linking its antimicrobial and anti-inflammatory properties with subsequent tissue repair. Where possible, specific honey types are linked to their characteristic or major bioactive constituents and to the experimental or clinical evidence supporting their use in particular wound settings. The review also considers medical-grade honey, emerging honey-based formulations, and the challenges of standardization and clinical translation. By integrating chemical, mechanistic, experimental, and clinical evidence, this review aims to clarify how the immunomodulatory properties of honey contribute to the regulation of wound inflammation and tissue repair, whether these effects differ among honey types, and what level of evidence currently supports their responsible use in modern wound management.

## 2 Methods

This review synthesizes current evidence on the antimicrobial, anti-inflammatory, antioxidant, and immunomodulatory roles of honey in wound healing. A literature search was performed in PubMed/MEDLINE, Web of Science, and Scopus for studies published between 1990 and 2025 using relevant keywords and MeSH terms. Additional studies were identified by screening reference lists and searching Google Scholar. Studies were included if they examined the antimicrobial, anti-inflammatory, antioxidant, immunomodulatory, or wound-healing effects of honey in in vitro, *in vivo*, or clinical settings. Editorials and studies without clearly defined interventions were excluded. Owing to variability in study designs and outcomes, the data were analyzed qualitatively, with focus on honey composition, mechanisms of action, and relevance to wound healing.

## 3 Composition of honey and its therapeutic variability across botanical sources

Honey is a natural product with a complex chemical composition that underlies its biological and therapeutic properties. More than 200 constituents have been identified, including carbohydrates, amino acids, enzymes, organic acids, vitamins, minerals, and a variety of bioactive phytochemicals [20], [21], [22] (Table 1). Carbohydrates constitute the largest fraction of honey, accounting for approximately 75–85 % of its composition. The main sugars are fructose (≈38–41 %) and glucose (≈31–34 %), together with smaller amounts of disaccharides such as sucrose and maltose and several oligosaccharides including erlose, maltotriose, nigerose, and kojibiose [23], 30], 31]. The high sugar concentration contributes to antimicrobial activity through osmotic effects, while the fructose–glucose ratio influences crystallization and physical stability, factors that may affect honey’s handling and use in wound dressings [32], 33].

**Table 1:** Bioactive compounds in honey and their primary roles in wound healing.

Bioactive compound	Primary mechanism(s)	Wound healing phase(s) targeted	Ref.
Glucose oxidase	Generates hydrogen peroxide (H_2_O_2_) upon dilution → sustained low-level antiseptic activity	Inflammation (antimicrobial control)	[22], 24]
Invertase, diastase	Facilitate sugar conversion; contribute to prebiotic-like oligosaccharide formation	General (microenvironment support)	[37]
Phenolic acids & derivatives	Scavenge ROS; inhibit NF-κB & cyclooxygenase (COX)-2; antimicrobial	Inflammation (antioxidant, anti-inflammatory), antimicrobial	[24], 41]
Flavonoids: chrysin, quercetin, galangin, pinocembrin, apigenin	Modulate immune cells & cytokines; inhibit matrix metalloproteinases (MMPs); promote angiogenesis & fibroblast proliferation; antioxidant	Inflammation (immunomodulation), proliferation (angiogenesis, matrix synthesis)	[37], 38], 42], 43]
Low molecular weight carbohydrates: fructose, glucose	Create hyperosmotic environment → dehydrating microbes; provide energy for fibroblasts & leukocytes	Inflammation (antimicrobial), proliferation (cellular energy)	[24], 32]
Organic acids: gluconic, acetic, butyric acid	Maintain low pH (3.4–6.1) → inhibit bacterial growth; antimicrobial contribution	Inflammation (antimicrobial)	[22], 37]
Antimicrobial peptides	Cationic peptides disrupt bacterial membranes; non-peroxide antibacterial activity	Inflammation (antimicrobial)	[24], 44]
Reactive carbonyls: methylglyoxal (MGO), DHA	Modify bacterial proteins & DNA non-enzymatically → cell death; major non-peroxide antibacterial factor in Manuka honey	Inflammation (antimicrobial)	[37], [38], [39]
Vitamins & minerals: ascorbic acid, B vitamins, zinc, copper	Cofactors in collagen synthesis (e.g., vitamin C); antioxidant defense; support cellular metabolism	Proliferation (matrix synthesis, cellular function)	[23], 34], 36], 45], 46]

In addition to sugars, honey contains water (approximately 10–20 %), small amounts of proteins and amino acids, vitamins, and minerals. Proline is the predominant amino acid and has been associated with collagen formation and tissue repair [34], 35]. Honey also provides several micronutrients, including vitamin C and B-complex vitamins, as well as minerals such as potassium, calcium, magnesium, iron, zinc, and manganese, which contribute to cellular metabolism and enzymatic activity [20], 23], 34], 36].

Several bioactive components are responsible for honey’s antimicrobial and wound-healing properties. When honey is diluted, the enzyme glucose oxidase becomes active and generates gluconic acid and hydrogen peroxide, which contribute to its antimicrobial action in wound environments [22]. Organic acids, including gluconic, citric, and malic acids, maintain an acidic pH (about 3.2–6.1), which inhibits microbial growth and may support fibroblast activity during tissue repair [22].

Polyphenols represent another important group of constituents contributing to honey’s biological activity. Flavonoids such as quercetin, kaempferol, apigenin, luteolin, chrysin, galangin, and pinocembrin, together with phenolic acids including gallic, caffeic, ferulic, syringic, and p-coumaric acids, contribute to antioxidant and anti-inflammatory effects. These compounds can modulate immune responses and influence cellular pathways associated with oxidative stress and inflammation [37], 38].

Certain honeys also contain distinctive antibacterial compounds. Manuka honey, for example, is characterized by high concentrations of MGO, which exhibits antibacterial activity independent of hydrogen peroxide by interfering with bacterial protein synthesis [39]. Experimental studies further suggest that honey may support tissue repair by maintaining fibroblast activity, modulating apoptosis, and promoting angiogenesis during wound healing [20], 22], 40].

The composition of honey varies according to its botanical and geographical origin. Monofloral honeys derived from a single plant species often show distinct phytochemical profiles. For example, Manuka honey contains high levels of MGO and dihydroxyacetone, whereas darker honeys such as chestnut are generally richer in phenolic compounds and minerals and tend to exhibit stronger antioxidant and anti-inflammatory activities [22], 38]. Environmental factors such as soil composition, climate, and regional flora also influence honey composition. Analytical techniques such as high-performance liquid chromatography and nuclear magnetic resonance are increasingly used to characterize these chemical profiles and support the standardization of honey intended for medical applications [46], [47], [48], [49]. Taken together, the diverse composition of honey helps explain its antimicrobial, antioxidant, anti-inflammatory, and wound-healing activities, which support its growing use as a natural therapeutic agent in modern wound management.

## 4 Cutaneous wound healing: cellular and molecular mechanisms

Cutaneous wound healing is a dynamic and context-dependent process that restores tissue integrity after injury. Although traditionally divided into four phases – hemostasis, inflammation, proliferation, and remodeling – these stages are highly overlapping and governed by continuous feedback between immune and structural cells (Figure 1). Increasing *in vitro* and animal studies indicate that disruption of this coordination, particularly at the level of inflammatory regulation, underlies chronic, and non-healing wounds [50], [51], [52], [53] (Table 2).

**Figure 1:**
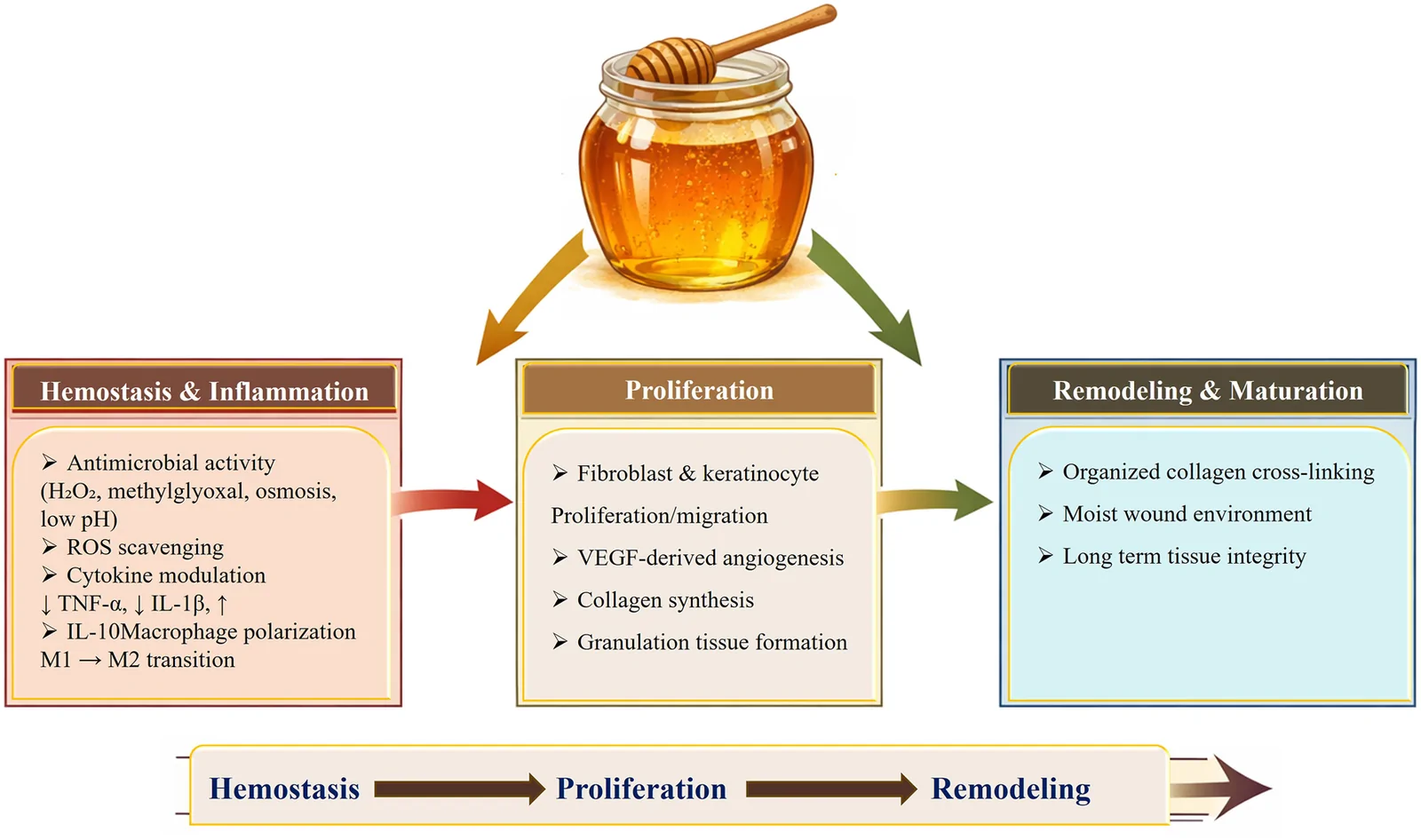
The multimodal therapeutic action of honey in the wound healing cascade. The timeline for the stages of healing a wound consists of the various phases of a wound healing process.

**Table 2:** Immunomodulators, cellular components, and molecular mechanisms across the four phases of cutaneous wound healing.

Phase	Key cells	Mediators (with functions)	Phase outcomes	Ref.
Hemostasis	– Platelets	PDGF – fibroblast recruitment, ECM synthesis. VEGF – vascular permeability, early angiogenesis signaling. FGF – fibroblast proliferation. TGF-β1 – ECM deposition and immune modulation. IL-1, TNF-α, IL-6 – initiation of inflammation. IL-8 – neutrophil recruitment. fibrin – clot scaffold formation. Fibronectin – cell adhesion and migration. von Willebrand factor – platelet adhesion.	– Clot formation – Immune cell recruitment – Initiation of inflammation	[59], 60]
Inflammation	– Neutrophils – M1 macrophages – Mast cells – Keratinocytes	HMGB1, S100, heat shock proteins – DAMP signaling. IL-1α, IL-33 – alarmin-mediated inflammation. TNF-α – endothelial activation. IL-8 – neutrophil chemotaxis. C3a, C5a – complement-mediated recruitment. ROS (H_2_O_2_) – antimicrobial activity of C–C motif chemokine ligand. (CCL)20, CCL27 – leukocyte recruitment.	– Pathogen clearance – Phagocytosis – Innate immune activation – Amplification of inflammation	[59], [60], [61]
Proliferation	– Fibroblasts – Keratinocytes – Endothelial cells – M2 macrophages – Regulatory T cells	PDGF – fibroblast proliferation. VEGF – angiogenesis. FGF – epithelial and fibroblast growth. TGF-β – collagen synthesis, myofibroblast differentiation. IL-10 – anti-inflammatory, resolution.	– Re-epithelialization – Angiogenesis – Granulation tissue formation – ECM deposition	[59], 60], 62]
Remodeling	– Fibroblasts (myofibroblasts)	Collagen III → collagen I – tensile strength increase. MMPs – ECM degradation and remodeling. TIMPs – inhibition of MMPs. ↓VEGF – vessel regression.	– Scar formation – ECM reorganization – Increased tensile strength – Wound contraction	[59], 60], 63]

### 4.1 Hemostasis

Hemostasis initiates immediately after injury and extends beyond a purely mechanical response. Platelet adhesion to exposed extra cellular matrix (ECM) components, including collagen, fibronectin, and von Willebrand factor, leads to clot formation through interactions with receptors such as glycoprotein VI [54], [55], [56]. The resulting fibrin clot, composed of platelets embedded in fibrin, fibronectin, vitronectin, and thrombospondin, not only prevents blood loss but also provides a provisional matrix and a source of bioactive signals [57], 58].

Several *in vitro* and animal studies showed that platelet degranulation releases mediators such as platelet factor (PF)4, platelet-derived growth factor (PDGF), vascular endothelial growth factor (VEGF), fibroblast growth factor (FGF), and transforming growth factor beta (TGF-β1), which regulate immune cell recruitment, angiogenesis, and early tissue responses. However, their effects are highly context-dependent; excessive PDGF or TGF-β1 signaling can promote fibrosis, whereas insufficient signaling impairs repair [64], [65], [66]. These studies also indicate that platelets initiate inflammatory cascades through cytokine release (e.g., IL-1α, IL-1β, IL-6, TNF-α, IL-8) and complement activation, generating C3a and C5a to enhance vascular permeability and leukocyte recruitment [50], 67], 68]. Thus, hemostasis and inflammation are functionally integrated rather than sequential. Despite advances, how early platelet-derived signals quantitatively shape downstream immune responses remains poorly defined, which may explain variability in platelet-based therapies [69], 70].

### 4.2 Inflammation

Inflammation is essential for pathogen clearance but represents a major point of dysregulation. It is triggered by damage-associated molecular patterns (DAMPs), ROS, calcium signaling, and lipid mediators released from damaged tissue. DAMPs such as high mobility group box 1 (HMGB1), S100 calcium-binding (S100) proteins, and ECM fragments activate pattern recognition receptors, including Toll-like receptors (TLRs), across immune and resident cells [71], [72], [73], [74].

Numerous *in vitro* studies showed that keratinocytes contribute actively to this phase through TLR expression (e.g., TLR3, TLR4, TLR9) and cytokine production [75], 76], although their relative contribution compared to immune cells remains unclear. ROS exemplify the dual nature of inflammatory mediators: they support antimicrobial defense but, when excessive, drive oxidative damage and impaired healing [77]. Macrophage plasticity is central to inflammatory resolution. While the classically activated macrophages (M1)/alternatively activated macrophages (M2) framework is widely used, it oversimplifies a continuum of activation states. The transition toward pro-repair phenotypes is critical for healing, yet the molecular determinants of this shift remain incompletely defined. Failure of this transition is strongly associated with chronic wounds [78].

### 4.3 Proliferation

The proliferative phase involves coordinated tissue reconstruction but remains tightly linked to preceding inflammatory signals. Angiogenesis, largely driven by VEGF and platelet-derived factors, restores perfusion [79], yet dysregulated angiogenesis may contribute to pathological outcomes.

Fibroblast activation under PDGF and TGF-β drives ECM deposition and granulation tissue formation, while differentiation into myofibroblasts enables wound contraction [80]. However, persistent fibroblast activation is a key driver of fibrosis, highlighting the narrow therapeutic window between repair and pathological remodeling.

Keratinocyte migration and proliferation restore the epidermal barrier through integrin-mediated interactions with the provisional matrix [81]. Impairment of these processes is a hallmark of chronic wounds but remains insufficiently targeted therapeutically. Increasing evidence points to the importance of fibroblast–macrophage crosstalk and integrin signaling in coordinating repair [54], 82], although their complexity complicates targeted intervention.

### 4.4 Remodeling

Remodeling is an active, long-term phase that determines tissue quality rather than simply concluding repair. Replacement of type III collagen with type I collagen and matrix reorganization are regulated by MMPs and tissue inhibitors of metalloproteinases (TIMPs) [54], 83]. Despite this control, regenerated tissue rarely recovers normal structure or function.

Fibrosis reflects a persistent imbalance between matrix synthesis and degradation, often driven by sustained TGF-β signaling and fibroblast activity [84]. While macrophages are increasingly recognized as regulators of this process [85], 86], therapeutic strategies targeting these pathways remain limited. Achieving functional, scar-free healing in adult tissues continues to be an unresolved challenge.

## 5 Integrated perspective and therapeutic implications

Wound healing is best understood as an interconnected network rather than a linear sequence. Early events in hemostasis shape inflammatory responses, which in turn determine the efficiency of tissue reconstruction and remodeling. Persistent inflammation and defective resolution are central features of chronic wounds [53].

This complexity limits the effectiveness of single-target therapies and supports the need for multimodal approaches that address inflammation, oxidative stress, and cell–cell interactions simultaneously. However, key gaps remain, including the regulation of macrophage heterogeneity, integration of mechanical and biochemical cues, and inter-individual variability in healing responses. Addressing these issues will be critical for advancing targeted and personalized wound therapies.

## 6 Honey-mediated immunomodulation and anti-inflammatory effects in wound healing

The beneficial effects of honey in wound healing extend beyond its antimicrobial activity. It helps maintain a moist wound environment, preventing tissue dehydration and supporting epithelial cell migration [87]. In addition, honey modulates the wound immune milieu during the inflammatory phase (Table 3). It influences immune cell activity, particularly macrophages and neutrophils, leading to attenuation of excessive inflammation while supporting the production of cytokines and growth factors required for tissue repair. Through these anti-inflammatory and immunomodulatory effects, honey contributes to a wound microenvironment that favors tissue regeneration and efficient healing [10], [88], [89], [90].

**Table 3:** Honey-mediated modulation of immunomediators in wound healing.

Immunomediator/process	Normal role in wound healing/inflammation	Effect of honey	Key mechanism(s)	Outcome in wound healing	Ref.
TNF-α	Pro-inflammatory cytokine; amplifies inflammation	↓ Downregulated	NF-κB inhibition; reduced cytokine expression	Reduced excessive inflammation	[88], 89], 91]
IL-1β	Early inflammatory cytokine	↓ Reduced	NF-κB suppression; pattern recognition receptors (PRR) modulation	Controlled inflammatory signaling	[88], 91]
IL-6	Acute inflammatory mediator	↓ Reduced	Downregulation of inflammatory signaling	Reduced inflammation intensity	[91]
IL-10	Anti-inflammatory cytokine; resolution phase	↑ Increased	Promotes M2 macrophage polarization	Resolution of inflammation	[49], 89], 92]
NF-κB pathway	Master inflammatory transcription factor	↓ Inhibited	IκB stabilization; blocked nuclear translocation	Broad suppression of inflammatory genes	[49], 92]
COX-1/COX-2	Prostaglandin synthesis enzymes	↓ Inhibited	Enzyme activity suppression	Reduced inflammation and pain	[93]
Prostaglandins (PGE2, prostaglandin F2 alpha, thromboxane B2 (TXB2))	Mediators of inflammation, pain, vascular effects	↓ Reduced	COX pathway inhibition	Reduced inflammatory symptoms	[94]
iNOS/NO	Antimicrobial; toxic at high levels	↓ Regulated	NF-κB inhibition; phenolic modulation	Reduced oxidative and inflammatory damage	[24], 41]
ROS	Microbial killing but causes tissue damage	↓ Scavenged	Antioxidant phenolics; Nrf2 activation	Reduced oxidative stress	[32], 95]
Nrf2 pathway	Cellular antioxidant defense system	↑ Activated	Keap1–Nrf2 activation → HO-1, NQO1, GST	Enhanced antioxidant protection	[33], 76]
M1 macrophages	Pro-inflammatory phenotype	↓ Suppressed	Reduced oxidative stress; immune modulation	Shift toward healing phenotype	[60]
M2 macrophages	Reparative, anti-inflammatory phenotype	↑ Promoted	IL-10; peroxisome proliferator activated receptor gamma (PPAR-γ), signal transducer and activator of transcription (STAT)6 activation	Tissue repair and regeneration	[60], 96]
Neutrophils	Early defense cells; excessive damage	↓ Controlled/apoptosis promoted	PRR signaling modulation	Reduced prolonged inflammation	[41], 88]
MMP-9	ECM degradation enzyme	↓ Regulated	Inflammatory pathway modulation	Improved ECM preservation	[97]
VEGF	Angiogenesis factor	↑ Supported	M2 macrophage polarization	Improved vascularization	[60]
Dendritic cells	Adaptive immune activation	↓ Modulated	Immune cell regulation	Controlled immune response	[97]
NF-κB–iNOS axis	Drives inflammatory NO production	↓ Inhibited	Phenolics (CAPE, quercetin)	Reduced inflammatory damage	[24]
MAPK pathways (ERK, JNK, p38)	Inflammatory signaling and cell survival	↓/Modulated	Polyphenol signaling regulation	Balanced inflammation and repair	[37]
NLRP3 inflammasome	IL-1β activation; pyroptosis	↓ Inhibited	Phenolic suppression	Reduced inflammatory cell death	[24]

A primary mechanism underlying honey’s anti-inflammatory activity involves modulation of pro-inflammatory cytokines and chemokines. *In vitro* and *in vivo* studies consistently demonstrate that honey downregulates the expression and secretion of key inflammatory cytokines, including TNF-α, IL-1β, and IL-6. When overexpressed, these cytokines perpetuate tissue damage, increase vascular permeability, and recruit excessive numbers of neutrophils [91]. Honey’s phenolic components, such as chrysin and caffeic acid phenethyl ester (CAPE), inhibit the NF-κB signaling pathway. NF-κB is a master transcriptional regulator of inflammatory genes; by preventing the degradation of its protein Inhibitor of κB (IκB) and subsequent nuclear translocation, honey limits the transcription of a wide array of pro-inflammatory mediators. Concurrently, honey enhances the production of anti-inflammatory cytokines such as IL-10, which plays a crucial role in resolving inflammation and promoting macrophage polarization toward the reparative M2 phenotype [49], 89], 92].

Additional *in vitro* evidence supports honey’s broad anti-inflammatory profile. Honey reduces the activity of COX-1 and COX-2 [93] and exhibits immunomodulatory effects [98]. Ingestion of diluted natural honey reduces plasma concentrations of prostaglandins, including prostaglandin E2, prostaglandin F2α, and thromboxane B2 in healthy individuals [89]. Bilsel et al. (2002) demonstrated that the anti-inflammatory activity of honey is comparable to prednisolone [99]. Notably, honey exerts anti-inflammatory effects without the adverse side effects typically associated with conventional agents. While corticosteroids may suppress immune function and impair tissue repair, and non-steroidal anti-inflammatory drug (NSAID)s are associated with gastric ulceration, honey avoids these complications [10]. Evidence indicates that honey attenuates inflammatory mediators while maintaining host defense, thereby reducing inflammation without systemic immunosuppression [41], 100].

In addition, honey supports key cellular processes essential for healing, including fibroblast proliferation, angiogenesis, and collagen synthesis [101]. Both experimental and clinical studies confirm that honey protects the gastric mucosa and does not induce ulcer formation, distinguishing it from NSAIDs and reinforcing its safety for prolonged use [89], 92]. Collectively, these findings highlight honey’s ability to provide effective anti-inflammatory activity without causing immune suppression, impaired tissue regeneration, or gastrointestinal toxicity.

Phenolic compounds present in honey can modulate nitric oxide (NO) production. These bioactive molecules influence the activity and expression of inducible nitric oxide synthase (iNOS) in macrophages and other immune cells, thereby regulating NO levels during inflammation. By modulating NO production and associated signaling pathways such as NF-κB and cytokine networks, honey polyphenols contribute to the control of inflammatory responses and support tissue repair processes. This immunomodulatory activity is considered one of the key mechanisms underlying the therapeutic effects of honey in wound healing and inflammatory conditions [102], [103], [104].

Nitric oxide functions as a multifunctional signaling molecule which affects wound healing through two different mechanisms: at elevated concentrations during the inflammatory oxidative burst, it becomes toxic to cells but at diminished constant concentrations it facilitates blood vessel expansion and new blood vessel development and fibroblast cell growth. The production of NO in response to honey shows different effects according to specific situations. Some studies suggest honey can reduce inducible nitric iNOS-mediated, excessive NO production in over-activated macrophages, thereby limiting tissue damage [41]. Phenolic compounds in honey, such as CAPE, strongly inhibit iNOS expression by interfering with NF-κB signaling. These compounds act as direct modulators of immune cell behavior by interacting with key cellular receptors and kinases. This interaction influences cell proliferation, apoptosis, and cytokine production, ultimately producing a coordinated therapeutic effect that regulates inflammation, oxidative stress, and immune responses [24] (Figure 2).

**Figure 2:**
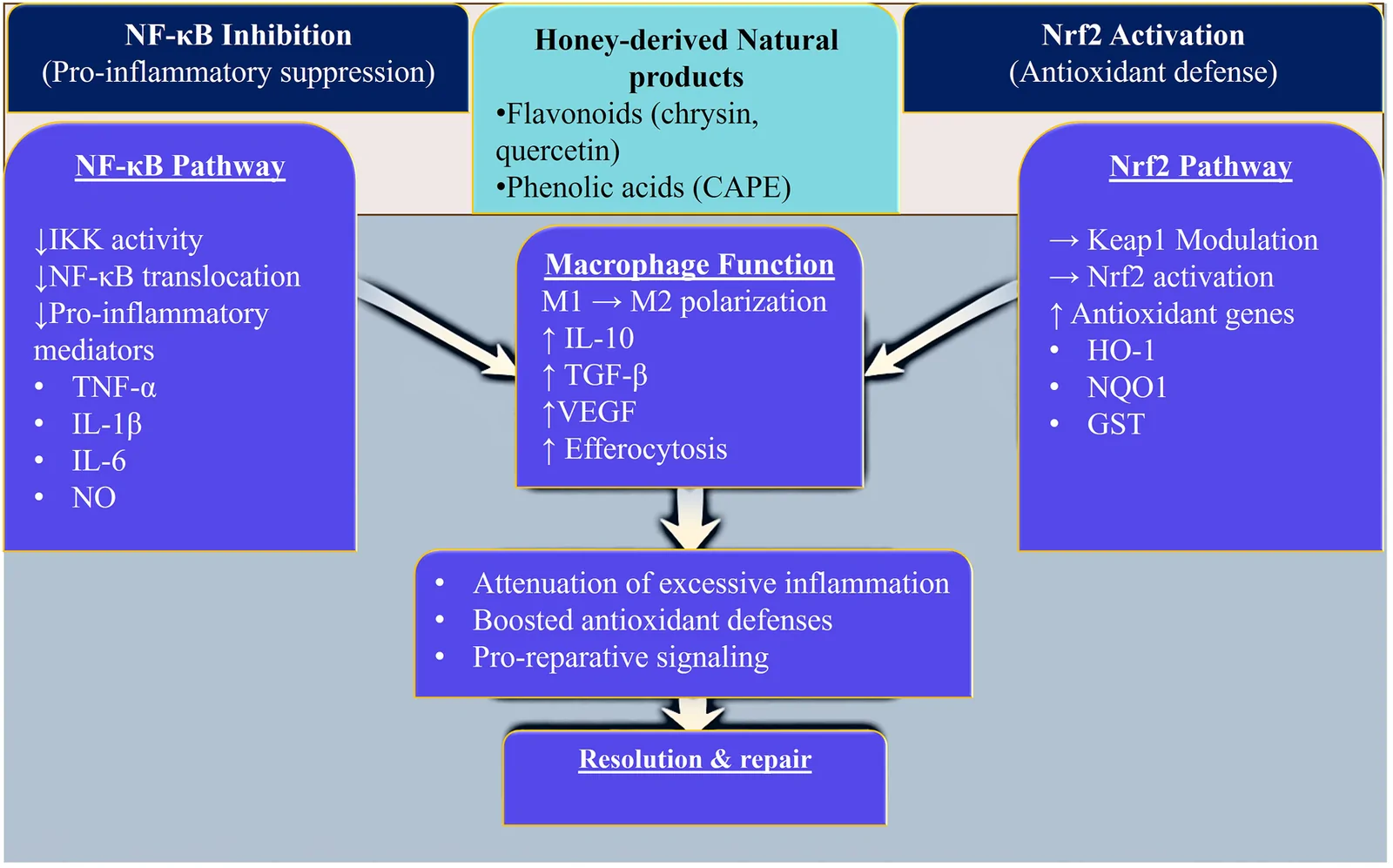
Molecular mechanisms of honey’s anti-inflammatory and immunomodulatory effects. This schematic illustrates how honey-derived natural products (flavonoids such as chrysin and quercetin, and phenolic acids such as CAPE modulate macrophage function in the wound microenvironment. On the pro-inflammatory M1 side, honey phenolics inhibit IKK activity, preventing NF-κB nuclear translocation and reducing transcription of TNF-α, IL-1β, IL-6, and iNOS. In parallel, honey activates the Nrf2 pathway by modifying kelch-like ECH-associated protein 1 (Keap1), releasing Nrf2 to translocate into the nucleus and induce antioxidant gene expression (HO-1, NAD(P)H quinone dehydrogenase 1 (NQO1), glutathione S-transferase (GST)). These combined effects promote macrophage polarization toward the M2 phenotype, characterized by enhanced transcription and secretion of IL-10, TGF-β, and VEGF, as well as increased efferocytosis of apoptotic neutrophils. Honey thereby attenuates excessive inflammation, boosts antioxidant defenses, and supports pro-reparative signaling, contributing to resolution of inflammation and tissue repair.

The phenolic compounds in honey provide immunomodulatory effects through multiple molecular targets. These include flavonoids such as chrysin, quercetin, galangin, and pinocembrin, as well as phenolic acids like caffeic acid, ferulic acid, and p-coumaric acid. These compounds can modulate NF-κB and Nrf2 signaling and also affect mitogen-activated protein kinase (MAPK) pathways, including extracellular signal-regulated kinase (ERK), c-Jun N-terminal kinase (JNK), and p38 mitogen-activated protein kinase (p38), which regulate both inflammatory gene expression and cell survival [37].

Certain honey phenolics have been shown to inhibit the nucleotide-binding oligomerization domain-like receptor protein 3 (NLRP3) inflammasome, a multiprotein complex responsible for IL-1β activation and pyroptotic cell death, further contributing to inflammation control [24]. The synergistic interactions among honey’s diverse phenolic constituents likely enhance their bioavailability and potency at the wound site, producing effects greater than the sum of individual components. Through these multifaceted mechanisms, honey emerges not as a simple dressing but as a sophisticated modulator of the wound immune landscape, capable of correcting the immunological imbalances that underlie healing failure [10].

Besides affecting inflammatory pathways like NF-κB, Nrf2, and MAPKs, the polyphenols in honey may also help resolve inflammation by influencing lipid mediator networks. Specialized pro-resolving mediators (SPMs), such as resolvins, protectins, and maresins, are part of these networks and actively reduce inflammation while promoting tissue repair [105]. Unlike conventional anti-inflammatory agents that broadly suppress immune responses, SPMs accelerate resolution by enhancing neutrophil apoptosis, promoting macrophage efferocytosis of apoptotic cells, and stimulating tissue regeneration [106]. While direct evidence for honey-induced SPM production remains limited, several lines of inquiry suggest potential connections. Honey’s polyphenolic compounds, particularly flavonoids such as quercetin and luteolin, have been shown to modulate arachidonic acid metabolism and may influence the biosynthesis of pro-resolving lipid mediators [42]. Additionally, Manuka honey’s anti-inflammatory effects in macrophage models share phenotypic similarities with SPM-mediated resolution, including reduced NF-κB activation, enhanced M2 polarization, and increased efferocytic activity [107]. Based on the above-discussed data, it is evident that honey exerts potent and coordinated anti-inflammatory effects that are central to efficient wound resolution. By attenuating excessive and prolonged inflammation, downregulating pro-inflammatory cytokines, reducing edema, and inhibiting prostaglandin synthesis, honey establishes a microenvironment conducive to tissue repair. These effects accelerate healing, limit the transition to chronic wounds, and contribute to pain reduction, without compromising cell viability or inducing cytotoxicity. Importantly, beyond its well-characterized anti-inflammatory properties, emerging evidence raises the possibility that honey may influence the active resolution phase of inflammation. Elucidating whether honey or its bioactive constituents can modulate SPM pathways, either by enhancing their biosynthesis or by engaging receptor-mediated signaling, represents a critical direction for future research. Confirmation of such mechanisms would distinguish honey from conventional anti-inflammatory agents, positioning it not only as a suppressor of inflammation but as a regulator of its timely resolution [105], [106], [107].

### 6.1 Honey as a modulator of oxidative stress in wounds

Honey also plays a critical role in the regulation of oxidative stress and ROS within the wound bed. While neutrophils generate ROS as a defense mechanism, an excessive and dysregulated oxidative burst leads to collateral damage of host tissues, lipid peroxidation, and impairment of fibroblasts and endothelial cells – a state known as oxidative stress [46]. Honey functions as a potent antioxidant due to its flavonoid and phenolic acid content, which can directly scavenge free radicals like superoxide anions and hydroxyl radicals. More importantly, components in certain honeys can upregulate endogenous antioxidant defense systems [95]. For instance, studies indicate that honey can activate the nuclear factor Nrf2 pathway. Upon activation, Nrf2 translocates to the nucleus and induces the expression of phase II detoxifying and antioxidant enzymes, such as heme oxygenase-1, NQO1, and GST. This endogenous upregulation provides a sustained cellular defense against oxidative insult, protecting nascent tissues and creating a redox-balanced environment conducive to proliferation [108].

Recent work with stingless bee honey demonstrated that its phenolic-rich fraction ameliorates oxidant–antioxidant imbalance through direct modulation of the Keap1–Nrf2 signaling axis, resulting in increased expression of HO-1 and NQO1 in diabetic rat models [76]. Similarly, natural Nrf2 activators derived from honey polyphenols have been shown to enhance antioxidant gene expression and reduce apoptosis in leukemic cell lines, underscoring honey’s potential as a chemopreventive agent [109]. These findings highlight that honey not only provides exogenous antioxidants but also potentiates intrinsic cellular defense machinery, thereby offering long-lasting protection against oxidative stress and supporting tissue regeneration (Table 3).

Based on the data discussed, honey modulates inflammation and immune responses in wounds, acting on macrophages, neutrophils, and other immune cells, while controlling oxidative stress and supporting tissue repair. Its phenolic and flavonoid compounds inhibit NF-κB and MAPK signaling, reduce pro-inflammatory cytokines, suppress excessive NO, and promote IL-10 production and M2 macrophage polarization. Nrf2 activation and possible effects on pro-resolving lipid mediators further enhance healing. Yet, most evidence is preclinical, and honey’s variable composition limits reproducibility and clinical translation. The specific contribution of individual mechanisms and the balance between reducing inflammation and preserving host defense remain unclear. Standardized formulations and rigorously designed clinical trials are needed to define honey’s therapeutic potential in wound care.

### 6.2 Immunomodulatory mechanisms of honey in coordinating wound healing

Honey exerts immunomodulatory effects that extend beyond simple suppression of inflammation, contributing to the regulation of both innate and adaptive immune responses during wound healing. Available evidence indicates that honey-derived sugars and phytochemicals interact with pattern recognition receptors (PRRs) on immune cells, leading to activation of monocytes and macrophages and initiation of an early inflammatory response [10], 43], 88], 110]. Importantly, this response appears to be transient and controlled, as reflected by the regulated production of cytokines such as TNF-α and IL-1β, which may support the resolution of impaired or chronic wounds rather than promoting sustained inflammation [88].

In later stages of healing, honey influences macrophage function by promoting the transition from the pro-inflammatory M1 phenotype to the reparative M2 phenotype. This shift is likely mediated by a combination of reduced oxidative stress and activation of signaling pathways such as PPAR-γ and STAT6 [60], 96]. M2 macrophages contribute to tissue repair through the production of anti-inflammatory cytokines, including IL-10, and growth factors such as VEGF, which support angiogenesis. In addition, honey has been reported to affect dendritic cell maturation, suggesting a role in modulating adaptive immune responses and limiting excessive immune activation at the wound site [60], 97].

Honey also affects neutrophil activity, promoting apoptosis and facilitating the clearance of cellular debris. These effects may help prevent prolonged inflammation and secondary tissue damage. At the same time, honey has been shown to modulate the activity of matrix metalloproteinases, particularly MMP-9, which are often elevated in chronic wounds and contribute to ECM degradation. By influencing inflammatory signaling pathways associated with MMP production, honey may help preserve tissue structure during repair [97].

Despite these findings, most of the available data are derived from *in vitro* or preclinical studies. Differences in honey composition, depending on botanical and geographical origin, together with the lack of standardized formulations, limit reproducibility across studies. Evidence from well-controlled clinical trials remains limited, and direct confirmation of these mechanisms in human wounds is still lacking. Further studies are needed to clarify the clinical relevance of these immunomodulatory effects [48], 88], 110].

## 7 Antimicrobial properties of honey in wound management

The potent and broad-spectrum antimicrobial activity of honey is a cornerstone of its therapeutic value in wound care, serving as a critical first step in creating a conducive environment for healing by controlling bioburden and preventing infection. Unlike many conventional topical antibiotics, honey employs multiple, synergistic mechanisms of action, which collectively reduce the likelihood of microbial resistance development. This multifaceted attack targets bacteria, fungi, and even some viruses, making it particularly effective against polymicrobial biofilms that commonly colonize chronic wounds [90].

The osmotic effect and low pH constitute fundamental physical-chemical barriers to microbial proliferation. Honey is a supersaturated sugar solution, creating a hyperosmolar environment when applied to a wound bed. This high osmotic potential draw lymph and interstitial fluid from the underlying tissues, effectively dehydrating microbial cells and inhibiting their growth. Concurrently, the natural acidity of honey, with a pH typically ranging from 3.4 to 6.1 primarily due to gluconic and other organic acids, further compromises bacterial viability. Most pathogenic bacteria thrive in a near-neutral pH (7.2–7.4); the acidic environment of honey disrupts their enzymatic activities and membrane functions, providing a simple yet effective inhibitory mechanism [16], 111].

A more dynamic and bioactive mechanism is the enzymatic production of H_2_O_2_. The enzyme glucose oxidase, introduced by bees during honey production, remains inactive in undiluted, stored honey due to low water activity. Upon application to a moist wound bed, the honey is diluted, activating the enzyme. Glucose oxidase then catalyzes the oxidation of glucose to gluconic acid and H_2_O_2_. The resulting steady, low-level release of H_2_O_2_ (typically in the micromolar range) acts as a potent non-selective oxidant, damaging microbial cell walls and DNA. Crucially, the concentration generated is often sufficient for bactericidal activity but low enough to avoid significant cytotoxicity to host fibroblasts and keratinocytes, a therapeutic window not easily achieved with exogenous H_2_O_2_ solutions. This endogenous, on-site production provides a continuous, mild antiseptic effect throughout the dressing period [15], 112].

Importantly, honey also contains Non-Peroxide Antibacterial Factors (NPAFs) that provide activity even when catalase (an enzyme present in some wound fluids) degrades H_2_O_2_. These are diverse and often specific to the floral source. The most studied is MGO, a highly reactive dicarbonyl compound that is exceptionally abundant in Leptospermum (Manuka) honey. MGO exerts its antibacterial effect by non-enzymatically modifying arginine and cysteine residues in microbial proteins and by cross-linking with bacterial DNA, leading to functional disruption and cell death. Other NPAFs include antimicrobial peptides like bee defensin-1, aromatic organic acids, and complex phytochemicals such as flavonoids and phenolic acids, which can disrupt bacterial cell membranes and potentiate the effects of conventional antibiotics [15], 39], 44].

The collective impact of these mechanisms on wound microbial load and biofilm control is profound. Honey has demonstrated efficacy against a wide range of clinically relevant pathogens, including MRSA, *P. aeruginosa*, and vancomycin-resistant enterococci [113]. Furthermore, it exhibits anti-biofilm properties. The osmotic effect and enzymes like glucose oxidase can help break down the extracellular polymeric substance matrix of biofilms, while components like MGO can penetrate and kill embedded, slow-growing bacteria. By reducing the bioburden and disrupting biofilm structures, honey mitigates the persistent inflammatory stimulus that pathogens provide, thereby indirectly supporting the transition from the inflammatory to the proliferative phase of healing [16], 114], 115]. This makes honey not merely an antiseptic but a strategic agent for modifying the entire wound microenvironment.

## 8 Clinical evidences supporting the use of honey in wound healing

Compared to the extensive *in vitro* and animal research, clinical studies investigating honey in wound healing are steadily increasing. Randomized controlled trials and systematic reviews indicate that honey, including standardized medical-grade formulations, can improve healing trajectories, reduce pain, and enhance patient-reported outcomes across several wound types. For example, meta-analyses of randomized controlled trials (RCTs) in obstetric wounds reported faster wound closure, lower analgesic requirements, and higher patient satisfaction with honey compared to placebo [29], 116]. While these results are promising, heterogeneity in study design, honey type, dosing, and outcome measures limits the generalizability of current clinical recommendations.

These clinical observations are grounded in a robust preclinical framework. *In vitro* studies demonstrate honey’s broad-spectrum bactericidal activity against key wound pathogens, including multidrug-resistant (MDR) strains such as MRSA and *P. aeruginosa*, as well as its ability to disrupt biofilms [117]. Laboratory assays consistently demonstrate honey’s broad-spectrum bactericidal activity against key wound pathogens, including MDR strains such as MRSA and *P. aeruginosa*, and its ability to disrupt biofilms [117]. Cell culture experiments have shown that honey promotes fibroblast and keratinocyte proliferation and migration, modulates macrophage cytokine profiles toward a pro-healing phenotype, and exhibits strong antioxidant properties [48], 118]. Complementary studies in animal models – such as rodent excisional, burn, and diabetic wound models – confirm that these effects translate into living systems. Across these models, honey consistently accelerates wound closure, enhances histological indicators of healing (including granulation tissue thickness, collagen deposition, and angiogenesis), and reduces both bacterial load and markers of inflammation compared with untreated or control-treated wounds [117], 118]. This robust preclinical evidence provides the biological rationale for conducting human trials (Table 4).

**Table 4:** Clinical evidence for honey in major wound types.

Wound type & level of evidence	Key clinical outcomes from RCTs/systematic reviews	Notes on honey type/formulation	Ref.
Partial-thickness burns-strong	Superior or comparable healing rates vs. conventional dressings. Significantly reduced time to epithelialization. Effective infection control and pain relief during dressing changes.	Mostly tested with Manuka honey-based dressings or gels; g-irradiated, medical-grade.	[119], [120], [121]
DFUs-moderate	Reduction in wound area, exudate, and odor. Effective against antibiotic-resistant pathogens. Mixed evidence on complete healing rates vs. advanced dressings; most benefit for infected or stagnant ulcers.	Various medical-grade honey (MGH) formulations used (algi); often Manuka or Revamil^®^ honey	[122], [123], [124]
VLUs – moderate/low	Enhanced debridement of slough and necrotic tissue. Reduction in wound size and inflammatory symptoms. Cochrane review found no clear difference in healing rates vs. other dressings, but noted benefit in debridement and infection control.	Used as primary or debridement dressing; various floral sources	[119], [120], [121], [122], [123], [124], [125]
PIs – emerging/moderate	Effective in reducing slough and promoting granulation tissue in Stages 2–4 ulcers. Helps manage exudate and odor.	Typically applied as a gel or impregnated in a dressing format	[125]
Acute traumatic & surgical wounds – moderate	Lower infection rates in contaminated wounds. Faster healing in infected post-operative wounds compared to antiseptic washes and conventional dressings. Useful for post-operative pilonidal sinus wounds.	Topical application or impregnated gauze	[119], 126]
Fournier’s gangrene (adjuvant therapy) – low	As an adjuvant to surgical debridement and antibiotics, associated with rapid infection control, reduced repeat debridements, and faster granulation.	Topically to wound bed post-debridement	[119], 126]

This table consolidates key findings from RCTs and systematic reviews on the efficacy of MG) for specific wound types, highlighting clinical outcomes and the strength of the supporting evidence.

Key for Level of Evidence and References: Strong: Consistent findings from multiple high-quality RCTs and/or a conclusive systematic review/meta-analysis. Moderate: Supported by several RCTs, but with some limitations (size, heterogeneity) or mixed findings on primary endpoints. Emerging/Low: Promising data from pilot studies, case series, or extrapolation from other wound types; larger, definitive RCTs are required.

The strongest evidence for honey in wound healing comes from clinical trials and systematic reviews. RCTs, the gold standard for clinical evaluation, have been conducted. A landmark Cochrane systematic review and meta-analysis by Jull et al. analyzed 26 RCTs comparing honey dressings with conventional dressings, silver sulfadiazine (AgSD), and other topical therapies for both acute wounds (burns, lacerations) and chronic wounds VLUs, DFUs, PIs) [119]. The review concluded, with moderate certainty, that honey dressings likely increase healing rates for partial-thickness burns and may improve outcomes for infected post-operative wounds compared with some conventional dressings.

Recent systematic reviews reinforce these findings. Bezerra et al. highlighted honey’s effectiveness in DFUs, while a 2024 review confirmed its benefits in burn wound healing compared with conventional treatments [127]. Clinical trials consistently demonstrate honey’s advantages in burn care, including shortened healing time, reduced hypertrophic scarring, and reliable antimicrobial control, often outperforming AgSD [127]. In DFUs, honey dressings significantly reduce wound size, odor, and exudate, and effectively manage antibiotic-resistant infections, although evidence regarding complete healing rates remains limited and warrants larger studies [127], 128]. These clinical benefits are largely attributed to reduced wound bioburden, modulation of inflammation, and maintenance of a moist, reparative wound environment.

However, critical appraisal reveals several limitations. A 2024 systematic review and meta-analysis evaluating honey dressings for chronic wounds – including DFUs, venous ulcers, and PIs – identified only eight studies meeting rigorous inclusion criteria, encompassing 906 patients [29]. While the analysis demonstrated significant acceleration in wound healing time (mean difference = −17.13 days, 95 % CI −26.37 to −7.89, *p* = 0.0003) and increased percentage of wound healing (MD = 18.31 %, 95 % CI 8.86 to 27.76, *p* = 0.0001), the overall quality of evidence was graded very low due to high risk of bias, heterogeneity, and publication bias [29]. No statistically significant differences were observed in complete healing rates (RR = 2.00, 95 % CI 0.78 to 5.10, *p* = 0.15) or bacterial clearance time [29]. Similarly, a systematic review of honey for episiotomy wounds reported beneficial effects on pain and healing, particularly when combined with adjuvant agents such as olive oil; however, methodological shortcomings – including small sample sizes, inadequate blinding, and variability in honey formulations – precluded definitive conclusions regarding efficacy [17]. These limitations highlight the need for larger, adequately powered RCTs with standardized honey preparations, consistent outcome measures, and extended follow-up to establish robust evidence for honey’s role in specific wound types [17], 29].

Beyond clinical efficacy, economic considerations are increasingly relevant given the substantial healthcare burden of chronic wounds. A recent RCT protocol comparing medical-grade honey with AgSD in burn care identified cost-effectiveness as a key outcome, hypothesizing that shorter hospital stays, reduced surgical interventions, and lower dressing costs could render honey an economically advantageous option [129]. Broader healthcare analyses suggest that even modest improvements in healing rates can yield significant cost savings at the population level [29]. Although the 2024 meta-analysis by Tang et al. found no significant reduction in hospital stay duration, trends toward faster healing and reduced infection may still translate into economic benefits in systems where prolonged treatment drives costs [29]. Moreover, honey represents a potentially cost-effective alternative in resource-limited settings where conventional advanced dressings may be unavailable or unaffordable [130]. Formal cost-effectiveness analyses incorporating direct and indirect costs, as well as quality-adjusted life years, are warranted to determine honey’s value relative to standard care [29], 129] (Table 3).

These clinical and economic findings underscore the therapeutic potential of honey across a range of wound types and highlight the importance of understanding the underlying biological mechanisms. Honey’s ability to modulate inflammation, regulate immune responses, and promote tissue repair suggests that its benefits extend beyond antimicrobial activity, emphasizing the significance of its anti-inflammatory and immunomodulatory properties in facilitating wound healing.

Preclinical studies provide strong mechanistic support for these clinical observations. *In vitro* investigations demonstrate honey’s broad-spectrum bactericidal activity against major wound pathogens, including MDR strains such as MRSA and *P. aeruginosa*, and its capacity to disrupt biofilms [117]. Honey also stimulates fibroblast and keratinocyte proliferation and migration, modulates macrophage cytokine profiles toward a pro-healing phenotype, and exhibits potent antioxidant activity [48], 118]. Complementary animal studies – including rodent excisional, burn, and diabetic wound models – confirm that these effects translate *in vivo*, accelerating wound closure, enhancing histological healing markers (granulation tissue, collagen deposition, angiogenesis), and reducing bacterial load and inflammation [117], 118], 131] Collectively, these findings provide a strong biological rationale for the observed clinical effects and support the integration of honey as a multifaceted therapeutic agent in modern wound management (Table 5).

**Table 5:** Comparative analysis of MGH and conventional topical antimicrobials in wound care.

Agent/characteristic	MGH	Silver dressings	Iodine-based dressings	Topical antibiotics	Ref.
Mechanism	Multi-target: osmotic, low pH, H_2_O_2_, bioactive compounds, immunomodulation	Disrupt microbial respiration	Oxidative damage via free iodine	Specific enzyme/protein synthesis inhibition	[88], 89], [131], [132], [133]
Spectrum	Very broad (bacteria, fungi, biofilms; incl. MRSA, vancomycin-resistant Enterococcus)	Broad (bacteria, some fungi)	Very broad (bacteria, fungi, viruses)	Narrow, pathogen-specific	[88], 89], 114], [131], [132], [133]
Key advantages	Anti-inflammatory; promotes healing & debridement; low resistance risk; safe	Strong antimicrobial; sustained release	Rapid bactericidal; good for exudative wounds	High efficacy for confirmed infections	[88], 89], [131], [132], [133], [134]
Key limitations	Variability; mild local irritation; not for severe systemic infection	Cytotoxic at high doses; may delay healing; resistance possible	Cytotoxic; thyroid concerns; irritation; systemic absorption risk	Resistance risk; dermatitis; limited spectrum; short-term use only	[13], [135], [136], [137], [138], [139], [140], [141]
Clinical role (TIME)	Debridement + inflammation/infection control; supports healing	Infection control (cautious use on clean tissue)	Infection control in heavily exuding wounds	Targeted, short-term infection treatment	[10], 88], [136], [137], [138]

TIME, tissue, infection/inflammation, moisture balance, edge advancement – a wound bed preparation framework.

## 9 Conclusions and future directions

Honey should be regarded as a biologically active therapeutic rather than merely a topical antimicrobial, with its anti-inflammatory and immunomodulatory properties playing a central role in wound healing. The available evidence indicates that honey modulates the wound immune microenvironment by limiting excessive inflammation while promoting its resolution, involving suppression of NF-κB signaling, activation of Nrf2-dependent pathways, and a shift in macrophage polarization from the pro-inflammatory M1 phenotype toward the reparative M2 state. These findings provide a mechanistic framework for its long-standing traditional use and link empirical observations to defined molecular and cellular processes. Such immunomodulatory effects are closely associated with enhanced angiogenesis, fibroblast function, and re-epithelialization, facilitating the transition of chronic wounds toward a pro-repair state.

Recent efforts have focused on the development of standardized, medical-grade honey with defined bioactive profiles. Future research should address key gaps, including the potential for tailoring specific honey types to wound characteristics and improving delivery through advanced systems such as nanofiber matrices and hydrogels, particularly in combination with other therapies. At the same time, there is a clear need for large, well-controlled randomized clinical trials using clinically relevant endpoints, together with omics-based approaches to better define the underlying molecular mechanisms and cellular responses.

Overall, medical-grade honey represents a promising multi-target approach for wound management, especially in conditions characterized by persistent inflammation and impaired healing. However, its broader clinical adoption remains limited by variability in composition, differences in botanical origin, lack of dose standardization, and weaknesses in study design. Many clinical studies remain small or methodologically limited, reducing the strength of the evidence base, and important aspects of its immunomodulatory and pro-regenerative mechanisms are still not fully defined. Addressing these limitations will be essential to support its integration into evidence-based wound care.
